# Supplementary material for: Modular cell-internalizing aptamer nanostructure enables targeted delivery of large functional RNAs in cancer cell lines
Source: Nat Commun. 2018 Jun 11;9:2283. doi: 10.1038/s41467-018-04691-x (PMC5995956; doi:10.1038/s41467-018-04691-x)
Supplement: Supplementary file 1 — Supplementary Information [file 41467_2018_4691_MOESM1_ESM.pdf]

Supplementary Information

Modular cell-internalizing aptamer nanostructure enables delivery of large functional RNAs in cancer cell lines

David Porciani[1,2,3], Leah N. Cardwell[1], Kwaku D. Tawiah[2,3], Khalid K. Alam[2,3,5], Margaret J. Lange[1], Mark A. Daniels[1] & Donald H. Burke[1,2,3,4]

[1]Department of Molecular Microbiology & Immunology, University of Missouri, Columbia, MO 65212, United States;
[2]Bond Life Sciences Center, University of Missouri, Columbia, MO 65211, United States;
[3]Department of Biochemistry, University of Missouri, Columbia, MO 65211, United States;
[4]Department of Bioengineering, University of Missouri, Columbia, MO 65211, United States;
[5]Department of Chemical & Biological Engineering, Northwestern University, Evanston, IL 60208 United States;

co-corresponding authors: burkedh@missouri.edu; porcianid@missouri.edu

Supplementary Table 1

	**Sequence 5' to 3'** (tail/anti-tail sequences are underlined)
**Waz**<br>**(2'F-Py RNA)**	GGGUUCUACGAUAAACGGUUAAUGAUCAGCUUAUGGCUGGCAGUU<br>CCCCGACGACGACGACGACGACGA
**C10.36**<br>**(DNA)**	CTAACCCCGGGTGTGGTGGGTGGGCAGGGGGGTTAGCGACGACGA<br>CGACGACGACGA
**3WJdB**<br>**(2'OH RNA)**	GGA*CCCACAUACUCUGAUGAUCCGAGACGGUCGGGUCCAGAUAU<br>UCGUAUCUGUCGAGUAGAGUGUGGGCUCGGAUCAUUCAUGGCAAG<br>AGACGGUCGGGUCCAGAUAUUCGUAUCUGUCGAGUAGAGUGUGG<br>GCUCUUGCCAUGUGUAUGUGGGUCGUCGUCGUCGUCGUCG<br>*GGA or GAA as first trinucleotide used to increase in vitro transcription yields.
**3WJtriB**<br>**(2'OH RNA)**	GGAUCCGCAUCAUCUAUCUGUCGAGUAGAGUGUGGGCUCCCCACA<br>UACUCUGAUGAUCCGAGACGGUCGGGUCCAGAUAUUCGUAUCUGU<br>CGAGUAGAGUGUGGGCUCGGAUCAUUCAUGGCAAGAGACGGUCG<br>GGUCCAGAUAUUCGUAUCUGUCGAGUAGAGUGUGGGCUCUUGCCA<br>UGUGUAUGUGGGGAGACGGUCGGGUCCAGAUAGAUGAUGCGGAU<br>UCGUCGUCGUCGUCGUCGUCG
**ctrl Apt**<br>**(non-targeted DNA sequence)**	GCCATTGCCATTGCCATTGCCATTGCCATTGCCATTGCCATTGCCAT<br>TGCCATTGCGACGACGACGACGACGACGA
**arbitrary DNA**[1]<br>**(Sgc8c DNA aptamer)**	ATCTAACTGCTGCGCCGCCGGGAAAATACTGTACGGTTAGATAGCGA<br>CGACGACGACGACGACGA
**arbitrary RNA**[2,3]<br>**(anti-CD4 2'F-Py RNA aptamer)**	GGGAGACAAGAAUAAACGCUCAAUGACGUCCUUAGAAUUGCGCAU<br>UCCUCACACAGGAUCUUUUCGACAGGAGGCUCACAACAGGCCGAC<br>GACGACGACGACGACGA

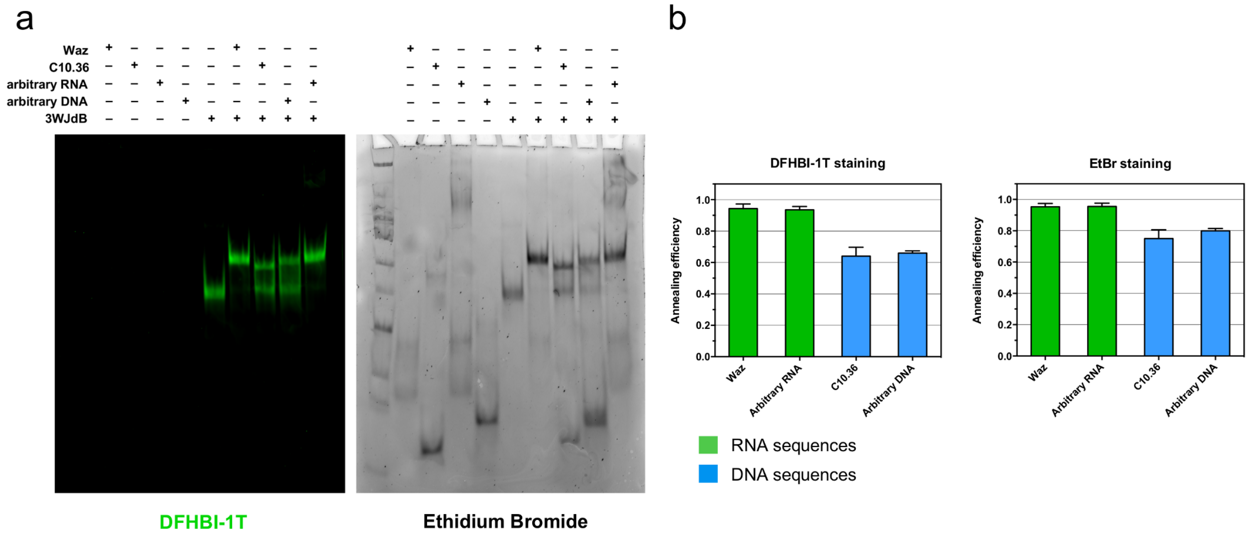

Supplementary Figure 1. Non-denaturing gel electrophoresis shows different annealing efficiency of RNA and DNA targeting sequences to 3WJdB
(**a**) 6% PAGE was stained first with DFHBI-1T and then with ethidium bromide. The intensity value of each band was estimated by densitometry analysis. The annealing efficiency for the annealed complex was calculated according to the following formula: [(annealed complex)/(free 3WJdB + annealed complex)]. Low range DNA ladder (GeneRuler™) was loaded in the first lane of the gel (**b**) Annealing efficiency values are plotted as the mean ± SD for three independent experiments. Waz and an arbitrary RNA with 2’F pyrimidines displayed almost 100% hybridization, independently from the staining approach used. On the other hand, annealing efficiency for C10.36 and an arbitrary DNA varies between 60-80% using DFHBI-1T and ethidium bromide staining, respectively. All sequences are listed in Table S1.

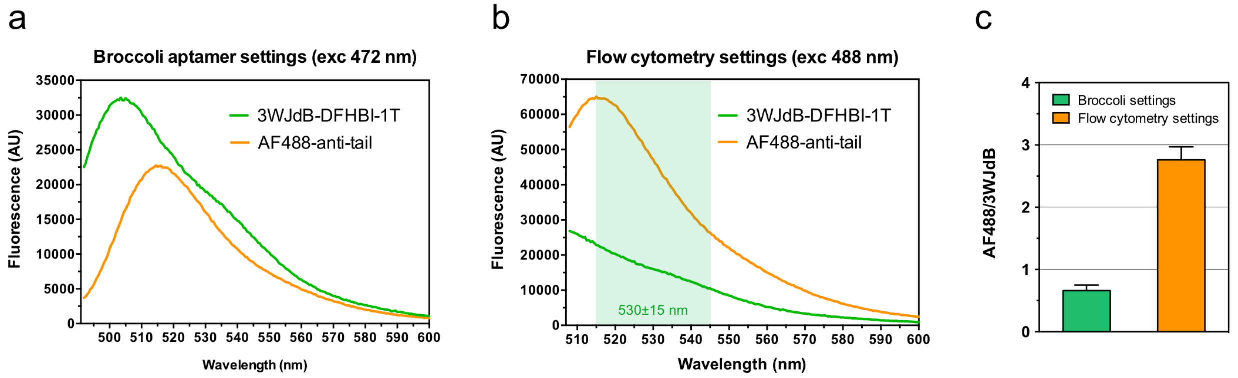

Supplementary Figure 2. Broccoli aptamer-DFHBI-1T fluorescence is suboptimal under standard flow cytometry settings

Standard flow cytometers utilize a blue light laser (488 nm) and a green emission filter (530/30 or 530±15 nm, green shaded box). These settings are well suited for GFP, AF488 or fluorescein isothiocyanate and similar fluorophores. In contrast, the fluorescence excitation maxima for RNA Broccoli-DFHBI-1T (472 and 507 nm) are shifted relative to these settings[4,5]. Therefore, we compared fluorescence of 3WJdB and AF488-anti-tail (0.5 μM) using conditions optimized for each. Fluorescence of 3WJdB (0.5 μM) was measured upon refolding in a buffer supplemented with DFHBI-1T (20 μM). (**a**) Emission from the 3WJdB (green) was ~1.5 fold brighter than AF488-anti-tail (orange) upon excitation at 472 nm ("Broccoli aptamer settings"). (**b**) In contrast, emission from AF488-anti-tail was ~3-fold higher than 3WJdB fluorescence upon excitation at 488 nm ("standard flow cytometry settings"). The difference between AF488 and 3WJdB in the latter conditions is easily explained: only the shoulder but not the peak of 3WJdB emission curve is collected. The non-optimal flow cytometry settings and the enhanced non-specific background observed in DFHBI-1T only-treated cells (see Figure 2, 3, and S12) led to an overall reduction of signal-to-noise ratio of 3WJdB. All curves are represented as averages of three independent experiments. **(c)** To calculate the ratio between fluorescence of AF488 and 3WJdB (histograms on the right) using the broccoli aptamer settings (green bar) and standard flow cytometry settings (orange bar), we first integrated the fluorescence area of each sample (from 495 to 600 nm in the "Broccoli aptamer settings" and from 508 to 600 nm in the "standard flow cytometry settings"), and then normalized for the fluorescence of 3WJdB for the respective settings. Values are the mean ± SD for three experiments.

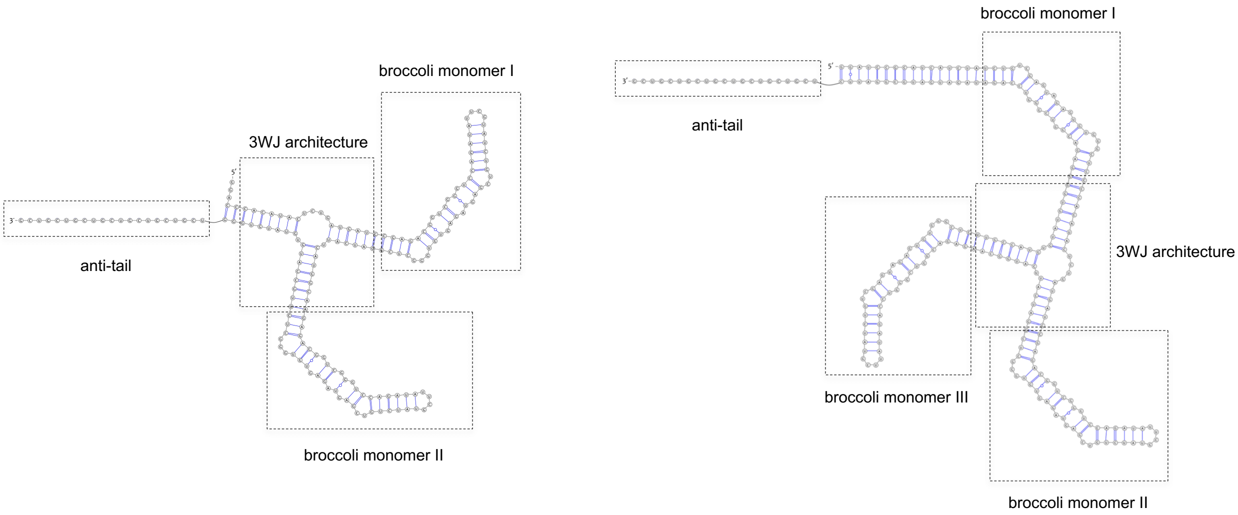

3WJdB-anti-tail (176nt, ~57kDa)

3WJtriB-anti-tail (244nt, ~79kDa)

Supplementary Figure 3. Secondary structure predictions of 3WJdB and 3WJtriB
NUPACK predicted secondary structure of 3WJdB and 3WJtriB were depicted using VARNA software[6]. The three main domains (3WJ scaffold, Broccoli aptamer unit, and anti-tail) that constitute these fluorogenic RNAs are indicated. All sequences are listed in Table S1.

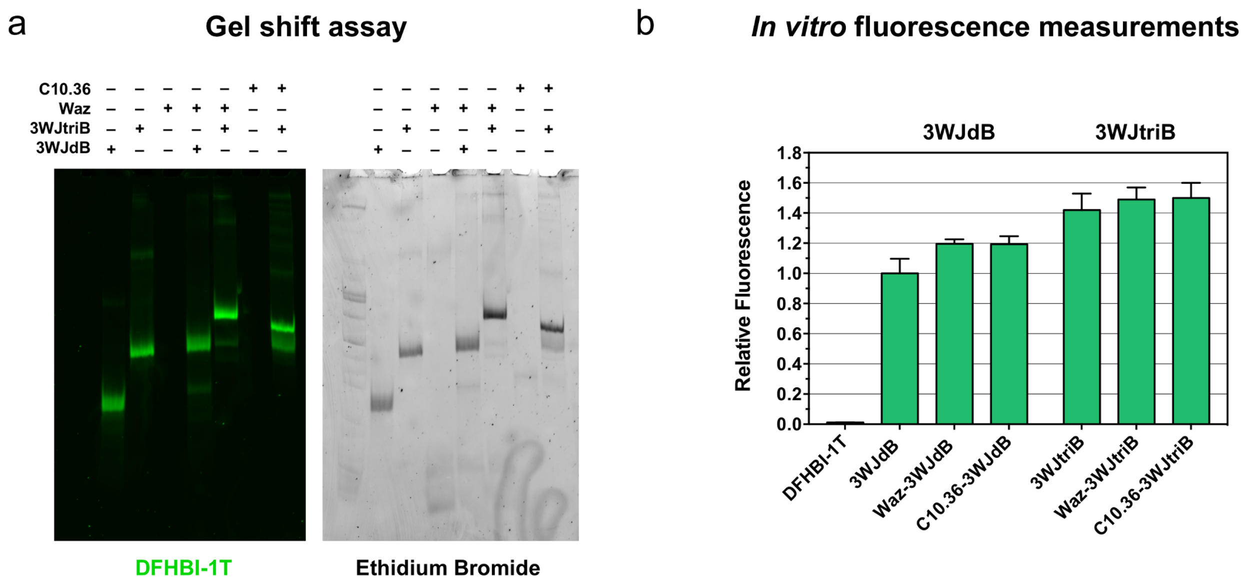

Supplementary Figure 4. Assembly and fluorogenic properties of aptamer nanostructures containing a trimeric version of Broccoli aptamer (3WJtriB)
(**a**) Hybridization between 3WJtriB and cell targeting aptamers was assessed by gel shift assay. 6% PAGE was stained first with DFHBI-1T and then with ethidium bromide. Bands with a reduced electrophoretic mobility appeared upon incubation of 3WJtriB with either Waz or C10.36, confirming effective formation of aptamer-aptamer hybrids. All sequences are listed in table S1. (**b**) Fluorescence spectroscopy in solution of free and assembled 3WJdB and 3WJtriB samples (λex = 472 nm; λem = 492-600 nm). Emission of 3WJdB or 3WJtriB (0.5 μM) was measured upon refolding in a buffer supplemented with DFHBI-1T (20 μM) either in absence or in presence of 3-fold molar excess of cell-targeting aptamers. Fluorescence of each sample was normalized for the fluorescence of free 3WJdB. Values are the mean ± SD for at least three independent experiments.

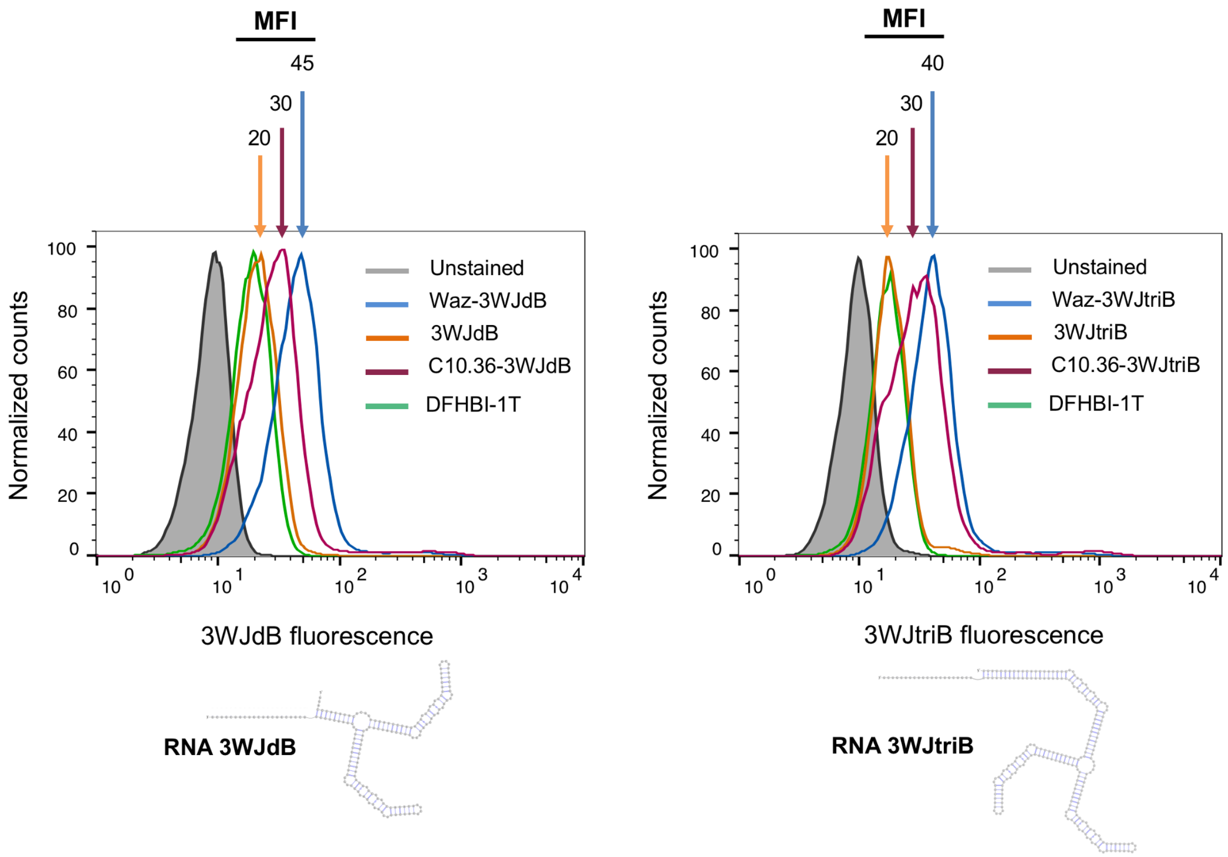

Supplementary Figure 5. Aptamer-mediated delivery of a larger fluorogenic RNA (244 nt) in NALM6 cells
3WJdB or 3WJtriB (0.5 μM) were assembled with 3-fold molar excess of cell-targeting aptamers Waz and C10.36, and binding to NALM6 cells was assessed after 1h incubation by flow cytometry. Representative flow cytometry curves illustrate a shift in fluorescence for aptamer nanostructures bearing both Waz (blue) and C10.36 (magenta) assembled with both 3WJdB (on the left) and 3WJtriB (on the right). DFHBI-1T only-treated cells (20 μM) are shown in green, and non-targeted controls (free 3WJdB or 3WJtriB) are shown in orange. Grey filled curve: unstained cells. Geometric mean fluorescence intensities (MFI) for each sample are shown above the respective curves. Normalized counts are reported on the *y*-axis, while on the *x*-axis is shown a log scale of fluorescence intensity. All curves are representative of two independent experiments.

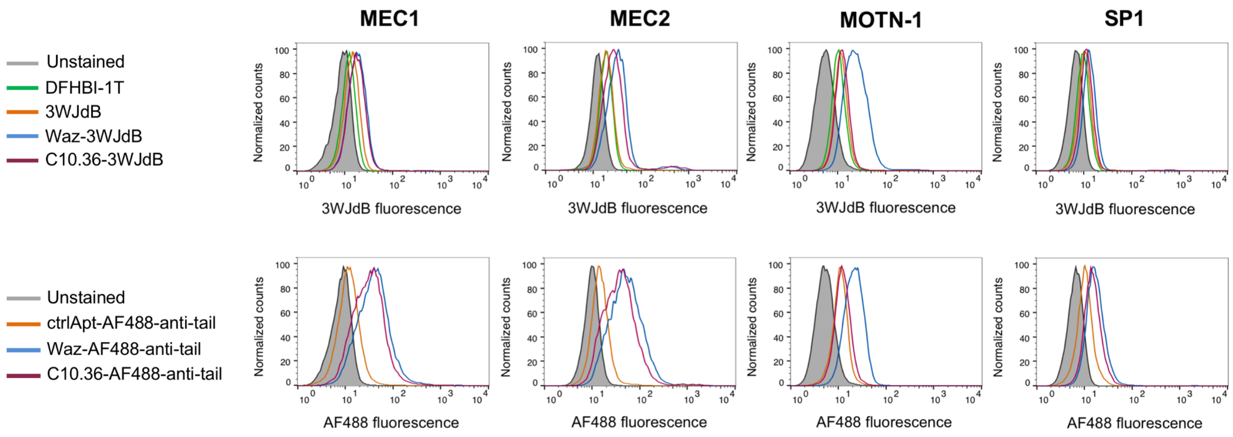

Supplementary Figure 6. Aptamer-mediated targeted delivery of large and small RNA payloads in leukemia cell lines

3WJdB or AF488-anti-tail (0.5 µM) were assembled with 3-fold molar excess of cell-targeting aptamers Waz and C10.36, and their relative controls. Targeting properties of the aptamer nanostructures were assessed toward two human B cell leukemia cell lines (MEC1 and MEC2), a human T cell leukemia cell line (MOTN1) and a mouse B cell leukemia cell line (SP1) as control. Cells incubated with 3WJdB samples were kept in cell-binding buffer supplemented with DFHBI-1T (20 µM) both during the 1h incubation and the flow cytometry analysis. Representative flow cytometry curves illustrate a shift in fluorescence for aptamer nanostructures bearing both Waz (blue) and C10.36 (magenta) assembled with both 3WJdB (top) and AF488-anti-tail (bottom). DFHBI-1T only-treated cells (20 µM) are shown in green, and non-targeted controls (free 3WJdB or a control aptamer assembled with AF488-anti-tail) are shown in orange. Grey filled curves: unstained cells. Normalized cell counts are reported on the *y*-axis, while on the *x*-axis is shown a log scale of fluorescence intensity. All curves are representative of three independent experiments.

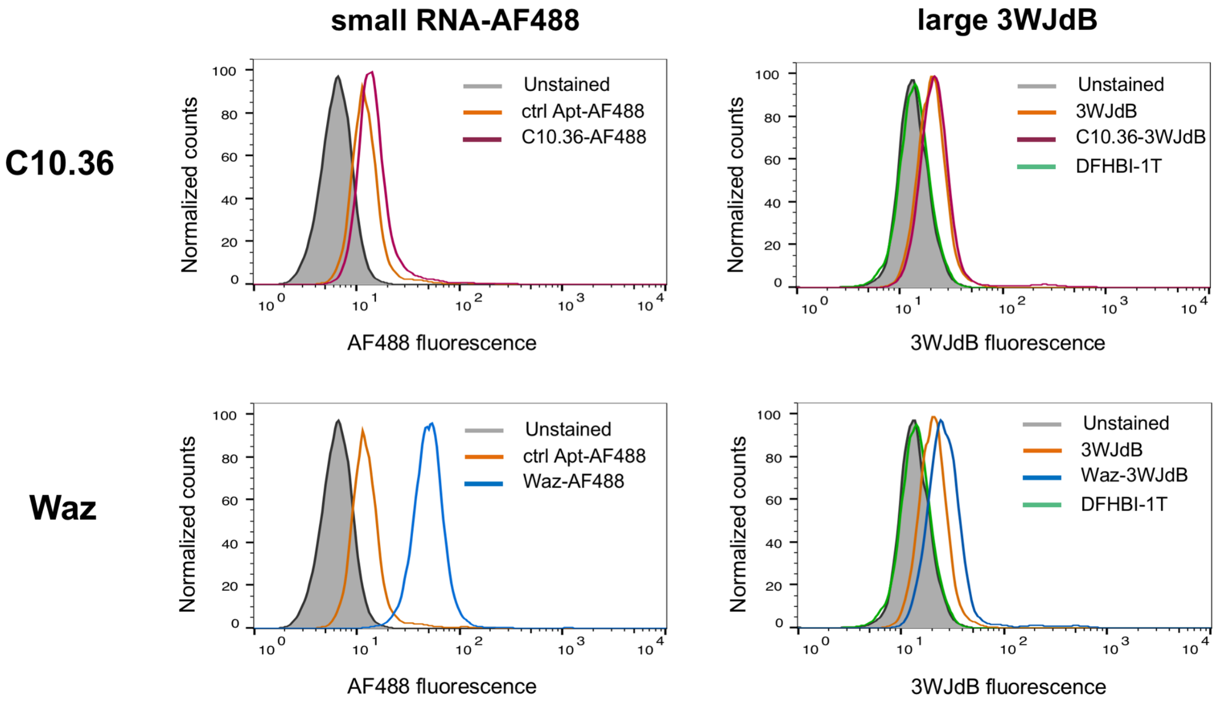

Supplementary Figure 7. Waz but not C10.36 is able to deliver both small and large RNA payloads in HeLa cells

A 3-fold molar excess of cell-targeting aptamers Waz and C10.36 and their respective controls were assembled with 0.5 μM of either AF488-anti-tail (on the left) or 3WJdB (on the right), and their abilities to bind HeLa cells were assessed after 1h incubation by flow cytometry. Representative flow cytometry curves illustrate a shift in fluorescence for both payloads only in presence of Waz (blue) but not C10.36 (magenta). DFHBI-1T only-treated cells (20 μM) are shown in green and non-targeted controls (free 3WJdB or ctrl Apt-AF488-anti-tail) are shown in orange. Grey filled curve: unstained cells. Normalized counts are reported on the *y*-axis, while on the *x*-axis is shown a log scale of fluorescence intensity. All curves are representative of three independent experiments.

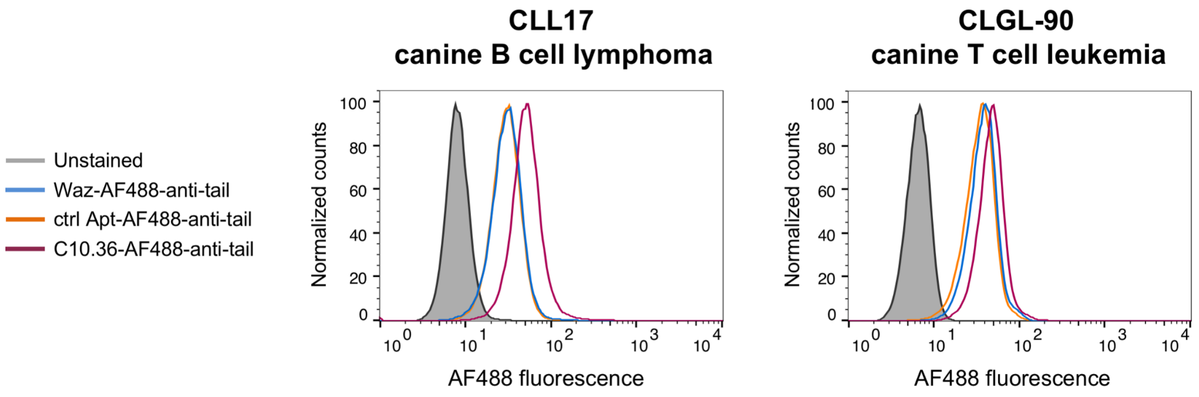

Supplementary Figure 8. Aptamer C10.36 specifically recognizes canine B cell lymphoma cell line

AF488-anti-tail (0.5 μM) was assembled with 3-fold molar excess of cell-targeting aptamers Waz and C10.36, and a control non-targeted aptamer, and their abilities to recognize canine lymphoma and leukemia cell lines were assessed after 1h incubation by flow cytometry. Representative flow cytometry curves illustrate a shift in fluorescence only in presence of C10.36 (magenta) but not Waz (blue). A control non-targeted aptamer loaded with AF488-anti-tail is shown in orange. Grey filled curve: unstained cells. Normalized counts are reported on the *y*-axis, while on the *x*-axis is shown a log scale of fluorescence intensity. All curves are representative of two independent experiments.

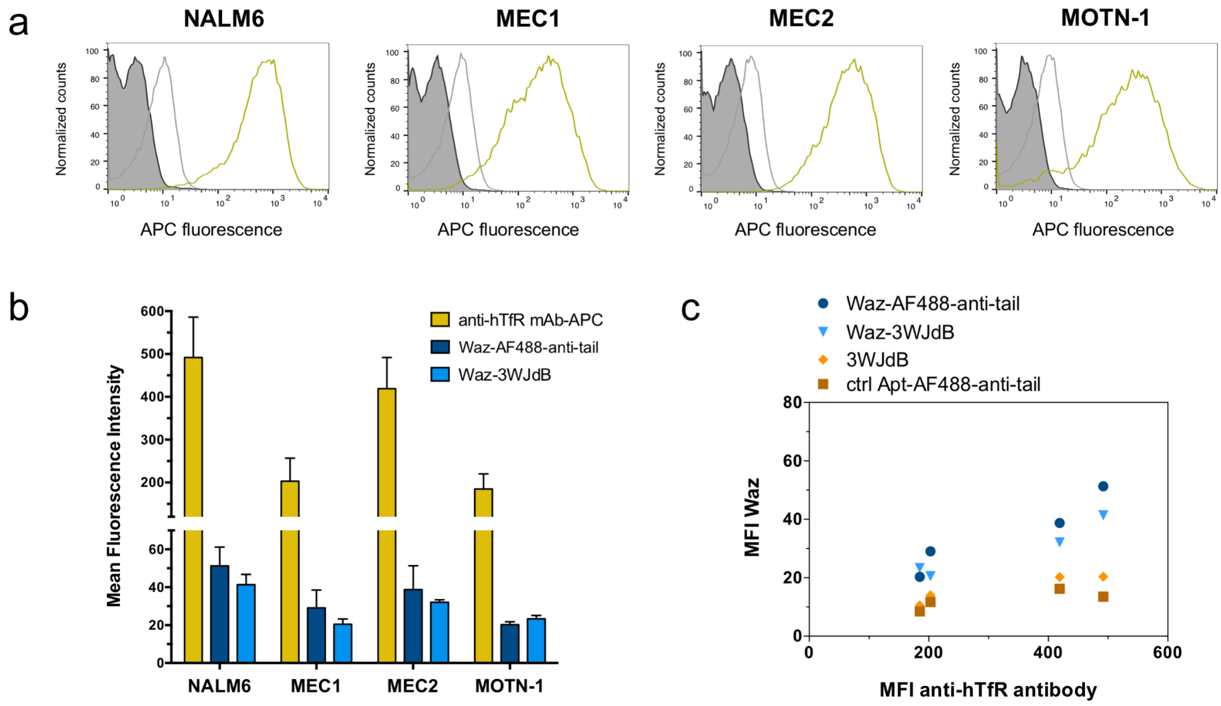

Supplementary Figure 9. Correlation between Waz cell-targeting properties and human TfR levels on target leukemia cells

An allophycocyanin (APC)-labeled anti-hTfR antibody was used to measure levels of hTfR expressed on three human B cell leukemia cell lines (NALM6, MEC1, MEC2) and a human T cell leukemia cell line (MOTN1). (**a**) Representative flow cytometry curves illustrate a shift in fluorescence due to the anti-hTfR antibody (gold) in all tested cell lines. Isotype antibody is shown in grey. Dark grey filled curve: unstained cells. (**b**) Comparison of mean fluorescence intensity measured in all four cell lines upon treatment with either anti-hTfR antibody (gold) or Waz (blue). MFIs for Waz samples are the same showed in Figure 2d and 2e. All values represent the mean ± SD for three independent experiments. (**c**) Scatter plot analysis correlates average MFIs of anti-hTfR antibody (*x*-axis) with the average MFIs of Waz-AF488-anti-tail (blue circle), Waz-3WJdB (blue triangle), free 3WJdB (orange diamond), and ctrl Apt-AF488 (brown square). Pearson's correlation coefficient for Waz-AF488-anti-tail and for Waz-3WJdB compared to the anti-CD71 antibody were r = 0.952 (P=0.048) and r = 0.961 (P=0.038), respectively. In contrast, correlation and p values for ctrl Apt-AF488-anti-tail and free 3WJdB were r = 0.785 (P=0.214) and r = 0.925 (P=0.075), respectively.

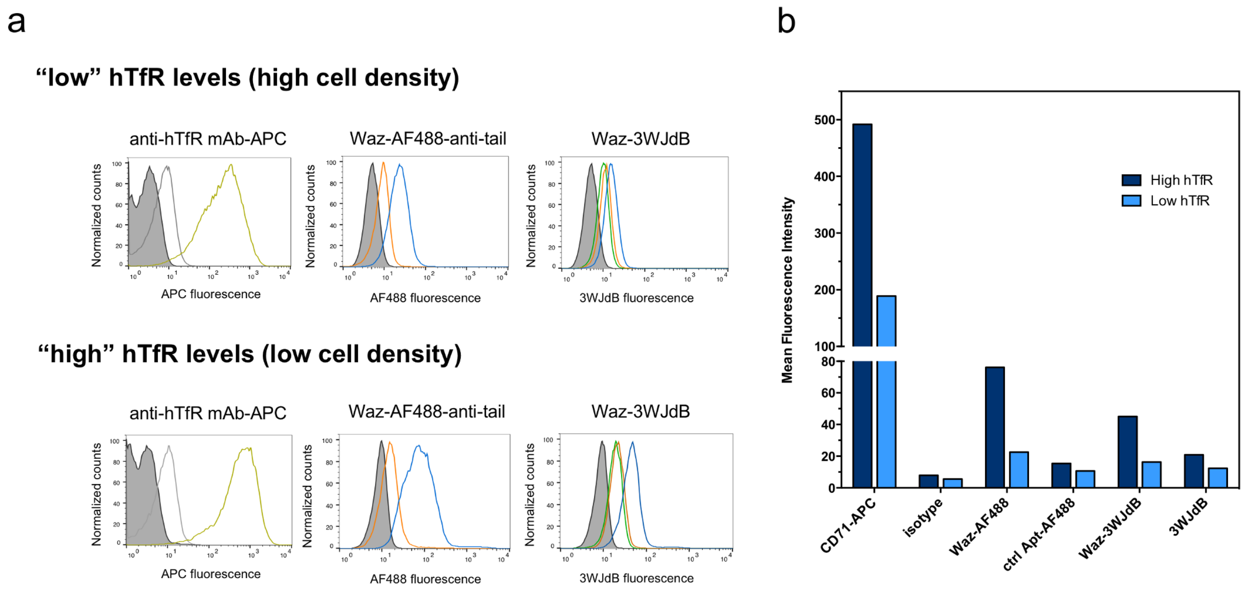

Supplementary Figure 10. Waz-dependent cell staining of NALM6 is sensitive to variations of human TfR levels
NALM6 cells were cultured at high density to reduce the expression of hTfR. An APC-labeled anti-hTfR antibody was used to measure levels of hTfR and its cell-staining was correlated with Waz-AF488-anti-tail and Waz-3WJdB. (**a**) Representative flow cytometry curves show that the magnitude of shift in fluorescence due to Waz samples (blue curves) positively correlated with the magnitude of shift obtained with the anti-hTfR antibody (gold curves). DFHBI-1T only-treated cells (20 μM) are shown in green and non-targeted controls (free 3WJdB or ctrl Apt-AF488-anti-tail) are shown in orange. Isotype antibody is shown in grey. Dark grey filled curve: unstained cells. (**b**) Mean fluorescence intensities of all samples measured at low hTfR levels (light blue bars) and high levels of expression (dark blue bars).

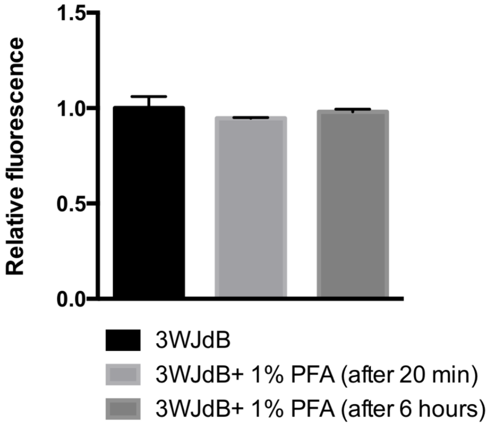

Supplementary Figure 11. 3WJdB-DFHBI-1T fluorescence is not affected by continuous treatment with PFA 1%
Upon refolding of 3WJdB aptamer in a buffer supplemented with DFHBI-1T, paraformaldehyde (PFA) was added to the sample up to a final concentration of 1% (v/v). Final concentrations of 3WJdB and DFHBI-1T in the solution including 1% PFA were 0.5 μM and 20 μM, respectively. A sample containing the same concentration of 3WJdB (0.5 μM) and DFHBI-1T (20 μM), but not PFA was used as reference sample (black bar). Fluorescence emission of 3WJdB was measured both after 20 minutes (light grey bar) and 6 hours (dark grey bar) of PFA treatment. Fluorescence of each sample was normalized for the fluorescence of untreated 3WJdB. Values are the mean ± SD for three independent experiments.

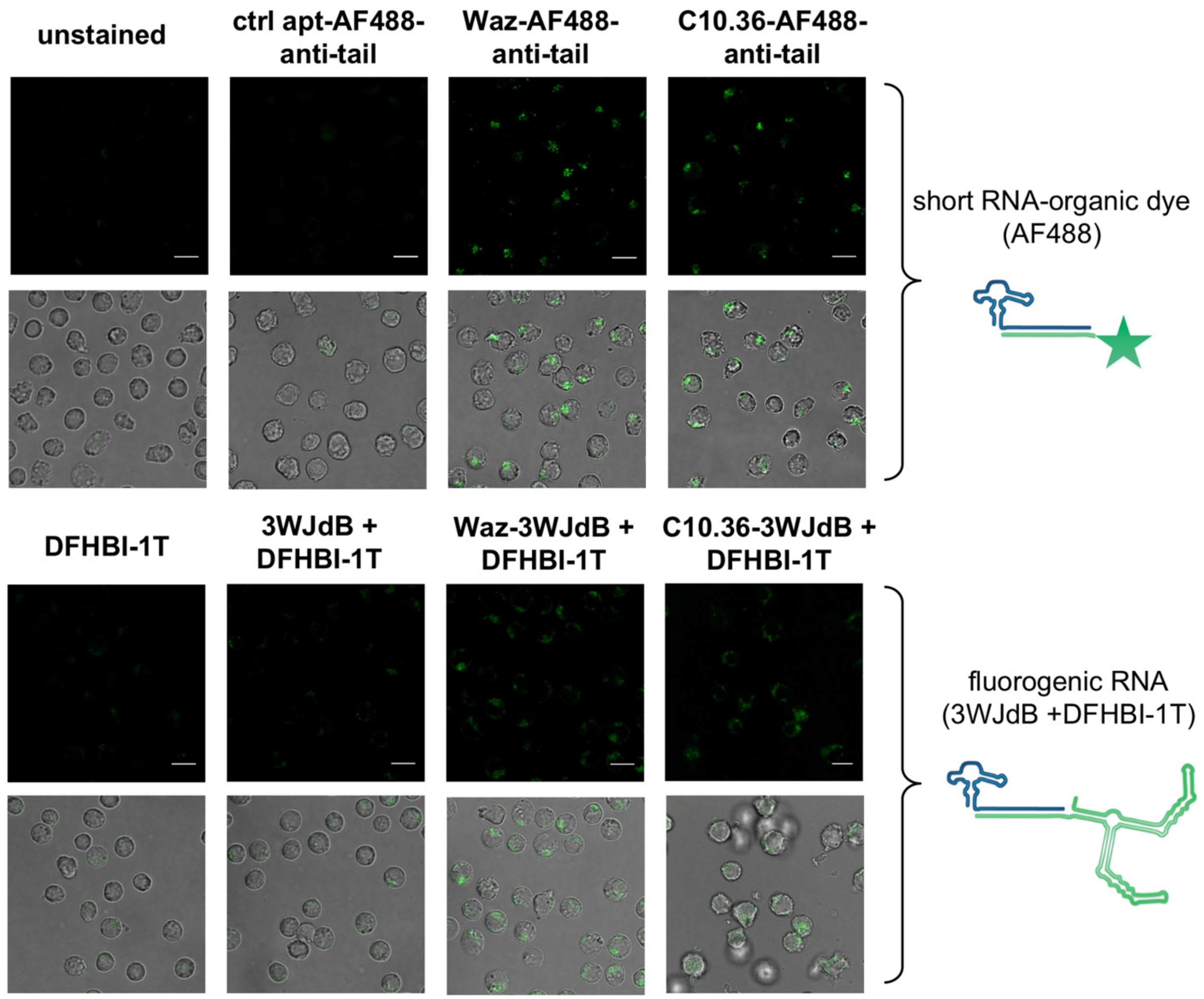

Supplementary Figure 12. Aptamer-mediated internalization of small and large RNA payloads in NALM6 cells monitored by confocal microscopy

Cell-targeting aptamers Waz and C10.36 and their respective controls were assembled with either 3WJdB or AF488-anti-tail (green), and their internalization properties were assessed after 1h incubation with NALM6 cells. Representative confocal microscopy images of fixed NALM6 cells show perinuclear vesicular signals (green) relative to both AF488-anti-tail and 3WJdB (0.5 μM) upon assembly with 3-fold molar excess of Waz and C10.36. In contrast, cells incubated with the non-targeted controls (control aptamer loaded with AF488-anti-tail or free 3WJdB) show minimal intracellular fluorescence. Cells incubated with 3WJdB samples were kept in cell-binding buffer supplemented with DFHBI-1T (20 μM) during both the 1h incubation and the confocal microscopy imaging. Compared with unstained cells, cells treated with DFHBI-1T only (20 μM) show a slight increase of fluorescence background, suggesting DFHBI-1T fluorescence activation via interaction with cellular components. Images are representative of three independent experiments. Scale bars: 10 μm.

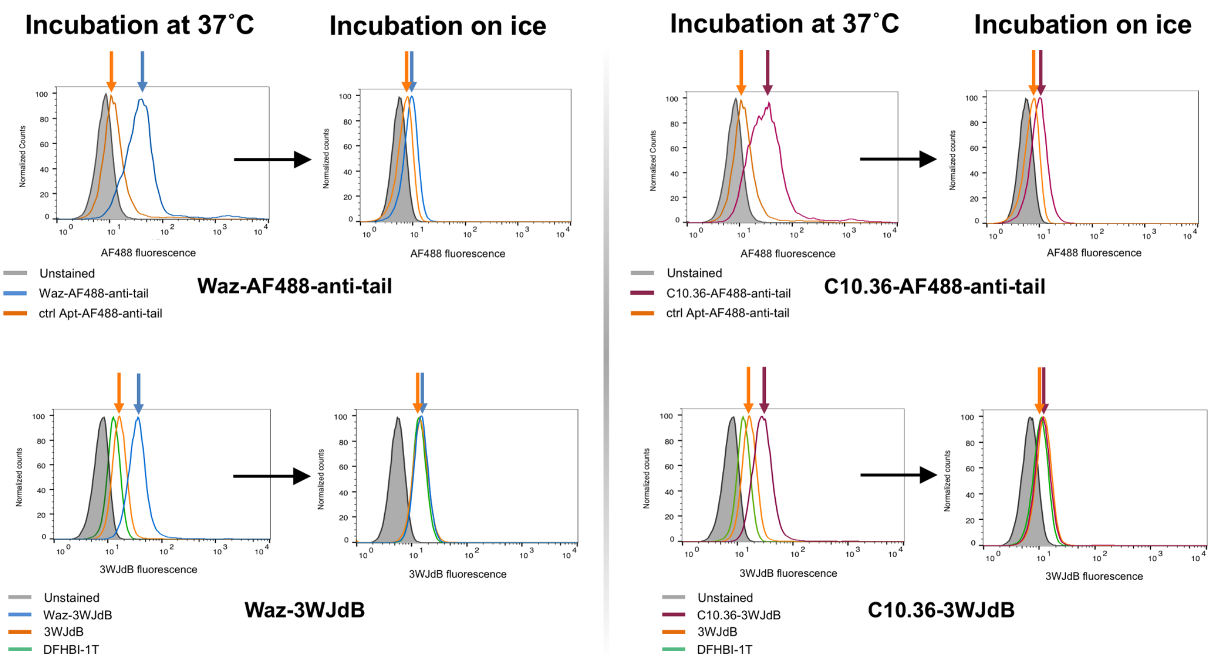

Supplementary Figure 13. An energy-dependent mechanism regulates aptamer-mediated cell staining of NALM6 cells

To arrest any energy-dependent mechanism of endocytosis, NALM6 cells were kept on ice for 10 minutes before incubation with aptamer samples. 3WJdB or AF488-anti-tail (0.5 µM) were assembled with 3-fold molar excess of cell-targeting aptamers Waz and C10.36, and their relative controls. Binding to NALM6 cells were assessed by flow cytometry after 1h incubation on ice. Representative flow cytometry curves illustrate a reduction of shift in fluorescence for both Waz samples (blue curves, on the left) and C10.36 samples (magenta curves, on the right) after incubation on ice. DFHBI-1T only-treated cells (20 µM) are shown in green and non-targeted controls (free 3WJdB or ctrl Apt-AF488-anti-tail) are shown in orange. Grey filled curve: unstained cells. Colored arrows that indicate geometric mean fluorescence intensity of Waz (blue), C10.36 (magenta) and non-targeted controls (orange) are shown above the respective curves under the two conditions of incubation (37°C and on ice). All curves are representative of two independent experiments.

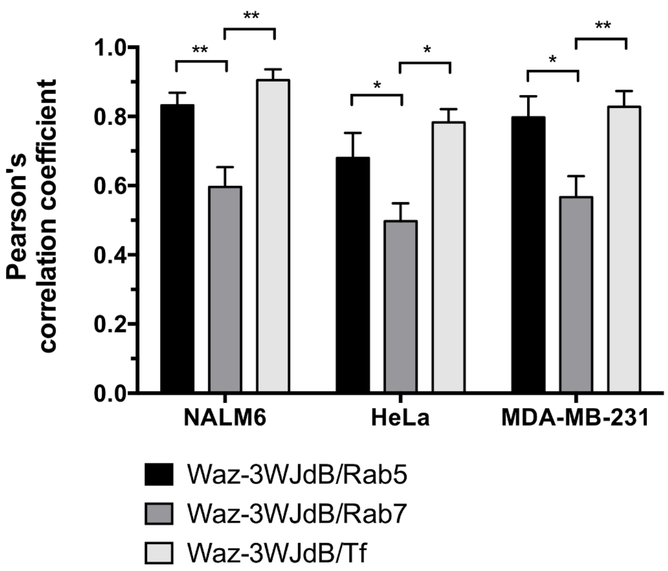

Supplementary Figure 14. Pearson's correlation coefficients between Waz-3WJdB-Cy3 and endocytic markers in three different cancer cell lines (NALM6, HeLa and MDA-MB-231)

Pearson's correlation coefficient was used to evaluate the coincidence detection between Waz-3WJdB-Cy3 and three endocytic markers (Rab5, Rab7, and Tf) in NALM6 cells (representative confocal microscopy images are shown in Fig. 4d, 4e, 4f), HeLa cells (representative confocal microscopy images are shown in Fig. 5) and MDA-MB-231 cells (representative confocal microscopy images are shown in Fig. S15). Values are the mean ± SD for two independent experiments. Statistical analysis for comparing multiple groups in each cell line was analyzed by one-way ANOVA with *post hoc* Tukey's test. Brackets with asterisks represent statistical difference: $^*p < 0.05$; $^{**}p < 0.01$

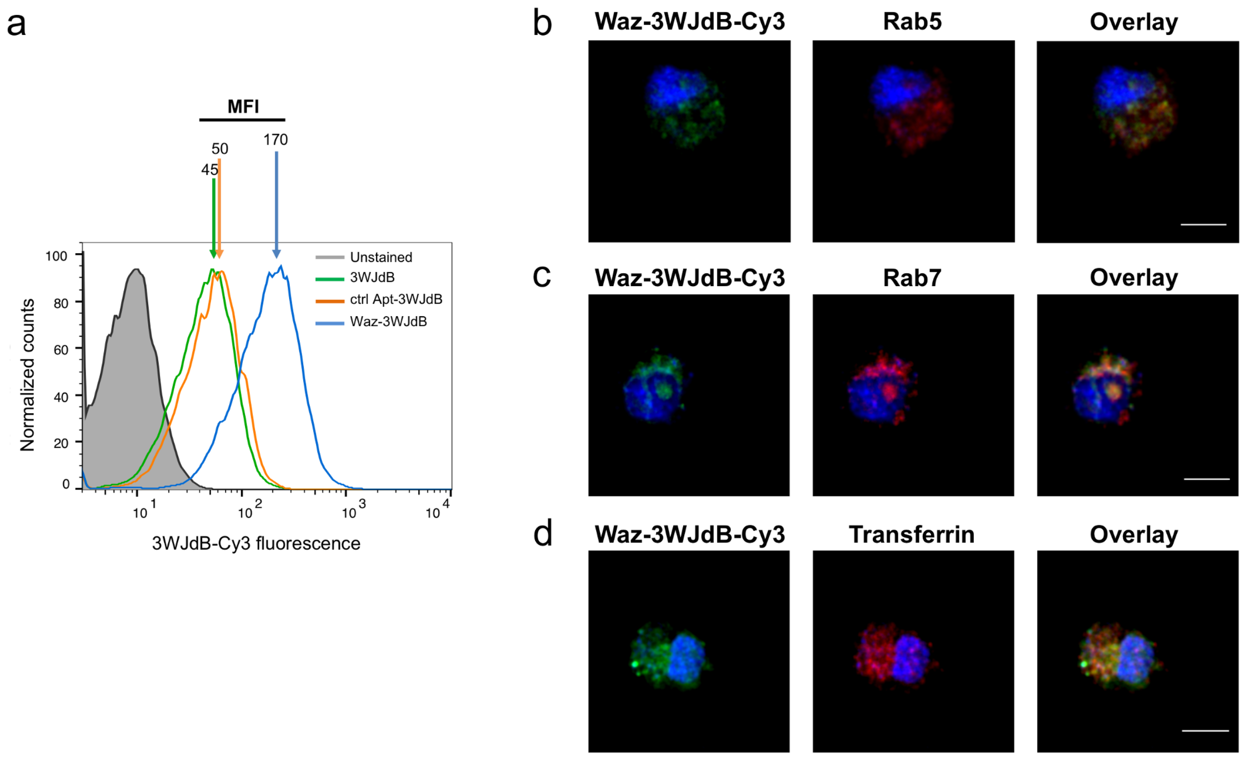

Supplementary Figure 15. Waz-3WJdB internalizes and localizes in the endosomes of MDA-MB-231 cells

(**a**) Cy3-labeled 3WJdB (0.5 μM) was assembled with 3-fold molar excess of Waz aptamer and a control non-targeted aptamer, and binding to MDA-MB-231 breast cancer cells was assessed after 1h incubation by flow cytometry. Representative flow cytometry curves illustrate a shift in fluorescence for Waz-3WJdB-Cy3 (blue) compared to controls (green and orange) and unstained cell (grey). 3WJdB-Cy3 alone (green), and ctrl Apt-3WJdB-Cy3 (orange) displayed the same magnitude of shift, indicating that the presence of a non-targeting sequence annealed to 3WJdB does not increase the extent of non-specific binding compared to 3WJdB alone. Geometric mean fluorescence intensities (MFI) for each sample are shown above the respective curves. Normalized counts are reported on the *y*-axis, while on the *x*-axis is shown a log scale of fluorescence intensity. All curves are representative of two independent experiments.
For colocalization studies, representative confocal microscopy images of fixed and immunostained MDA-MB-231 cells are shown on the right; Cy3-labeled 3WJdB (green) was assembled with 3-fold molar excess of Waz aptamer, and colocalization with three different endocytic markers (red) (Rab5, Rab7, and Tf) was assessed after 1h-incubation in MDA-MB-231 cells. (**b**) We observed significant colocalization between Waz-3WJdB-Cy3 and Rab5 (early endosome marker). (**c**) A reduction of colocalization was found between Waz-3WJdB-Cy3 and Rab7 (late endosome marker). (**d**) MDA-MBD-231 cells were co-incubated for 1h with 0.5 μM AF488-labeled Tf and 0.5 μM Waz-3WJdB-Cy3 complex, then cells were fixed and imaged by confocal microscopy. A strong colocalization between Tf-AF488 and Waz-3WJdB-Cy3 was observed. For all samples, Pearson's correlation coefficient was used to estimate the extent of colocalization between Waz-3WJdB-Cy3 and endocytic markers (see Figure S14). Images are representative of two independent experiments. Scale bars: 5 μm.

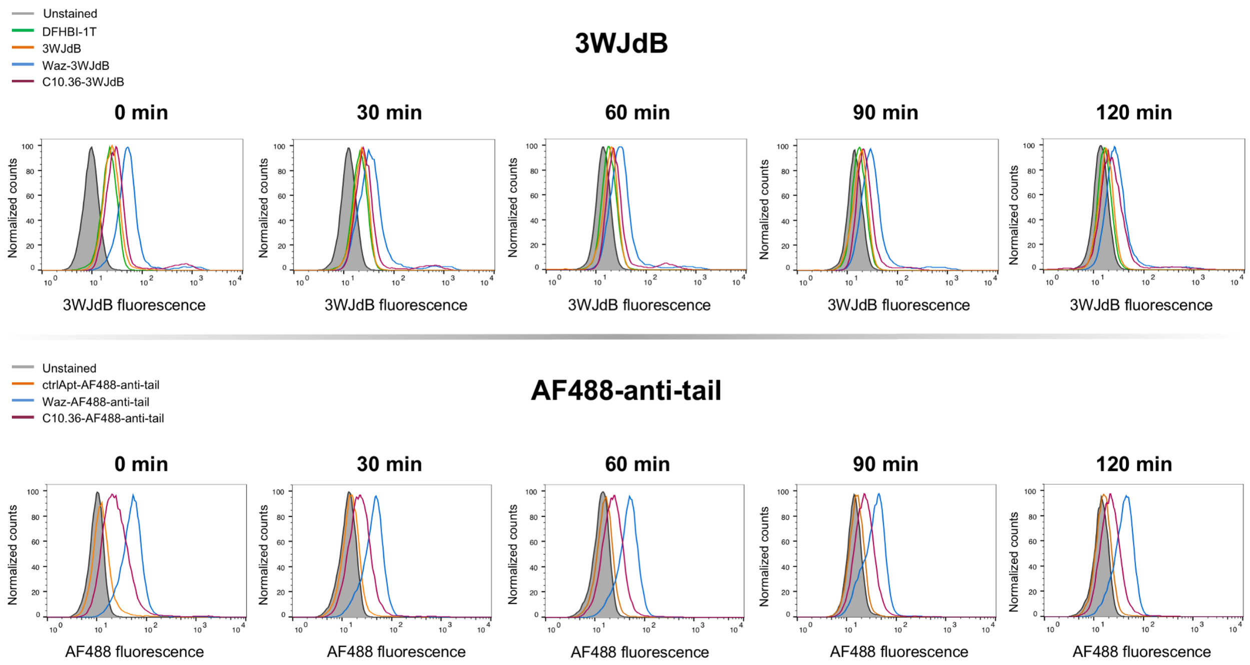

Supplementary Figure 16. Flow cytometry analysis for studying persistence of 3WJdB fluorescence following aptamer-mediated delivery in B cell leukemia cells

NALM6 cells were incubated with aptamer samples for 1 hour (pulse). Then, cells were washed and incubated for an additional 120 minutes in aptamer-free medium (chase). Samples containing 3WJdB were kept in a medium supplemented with DFHBI-1T (20 μM) during the 1h incubation (pulse phase), the entire chase phase (120 minutes), and the flow cytometry analysis. Representative flow cytometry curves illustrate a shift in fluorescence for aptamer nanostructures bearing both Waz (blue) and C10.36 (magenta) assembled with both 3WJdB (on the top) and AF488-anti-tail (on the bottom). DFHBI-1T only-treated cells (20 μM) are shown in green, and non-targeted controls (free 3WJdB or a control aptamer assembled with AF488-anti-tail) are shown in orange. Grey filled curve: unstained cells. All curves are representative of three independent experiments.

Supplementary References

1. Shangguan, D., Tang, Z., Mallikaratchy, P., Xiao, Z. & Tan, W. Optimization and modifications of aptamers selected from live cancer cell lines. *Chembiochem* **8,** 603–6 (2007).
2. Davis, K. A., Lin, Y., Abrams, B. & Jayasena, S. D. Staining of cell surface human CD4 with 2’-F-pyrimidine-containing RNA aptamers for flow cytometry. *Nucleic Acids Res* **26,** 3915–3924 (1998).
3. Wheeler, L. A. *et al.* Inhibition of HIV transmission in human cervicovaginal explants and humanized mice using CD4 aptamer-siRNA chimeras. *J. Clin. Invest.* **121,** 2401–2412 (2011).
4. Song, W., Strack, R. L., Svensen, N. & Jaffrey, S. R. Plug-and-play fluorophores extend the spectral properties of Spinach. *J. Am. Chem. Soc.* **136,** 1198–201 (2014).
5. Ouellet, J. RNA Fluorescence with Light-Up Aptamers. *Front. Chem.* **4,** 29 (2016).
6. Blin, G., Denise, A., Dulucq, S., Herrbach, C. & Touzet, H. VARNA: Interactive drawing and editing of the RNA secondary structure. *IEEE/ACM Trans. Comput. Biol. Bioinforma.* **7,** 309–322 (2010).
